# The Stomach

**Published:** 1869-10

**Authors:** 


### THE STOMACH.

### Æsthetically Considered.

BY ISHMAEL.

Everybody has one.

But the amusing part of it is, that few know anything about it. They carry it before them all their days; it is under their very eye, as it were, as long as they live; but taken away from them—brought forward to be introduced and to be exhibited as showing how it literally "works for their living," each one would exclaim, "Pooh!" and add, "I have nothing of that sort about *me!*"

I live, sandwiched, as one might say, between two Doctors, a member of the medical profession being my nearest neighbor on either hand; which will account for the immense physiological knowledge I am about to display.

Outside of medical works and patent medicine advertisements, the subject of our article is seldom referred to in our literature, and when it is, it is called by an unpleasant word that rhymes with jelly. Clement Moore, in his "Night before Christmas," thus ignorantly uses the word to describe the rotund stomach of jolly Santa Claus; Washington Irving, too, introduces it tenderly and affectionately in his description of the personal appearance of the waddling heroes that governed New Netherlands, while Shakespeare, in one or two instances, readily called to mind, alludes to it.—Whether this slighting of an important organ, indeed, the most important of our system, arises from an ignorance of its worth and its capabilties, or that it is too delicate to be lightly referred to; or because, like the lower classes, it does all the hard work of the body and so is vulgar, or because it is strong enough to care for itself, is a matter that I have been wholly unable to determine. But it is very true that the love-sick swain, sighing and pressing his hand upon his heart; that the scholar, appealing to his brains and his head; and even the prize-fighter, looking to his lungs and his muscle, all depend more upon their stomachs than upon the parts to which they directly appeal for their several conditions or success.

At best, it is a singular distribution of the affairs of this world, that whatever is most excellent and most desirable, is applied, if not to evil and disreputable purposes, at least to those that are disagreeable and unpleasant. The most delicious music that is written, is so associated with the lewd and lascivious that the one cannot be separated from the other; the most pleasing and fascinating games are so connected with and surrounded by evil associations that they have come to seem part and parcel of each other, and the stomach has so long been associated with epicureanism, dainties, famous cooks, tid-bits and luxuries, that to speak of one invaribly suggests the other.

It is a strong, helpful, working, earnest, patient servant of ours, this stomach, and since it has been so thoroughly misused, the only wonder is that men have any stomachs at all. Certain it is, that if of other servants we exacted as much as we do of this, they would leave without warning.

I know nothing more like in shape to the stomach, than the pouch of a bag-pipe, with its upper side concave, its lower convex. It has three coats, and it deserves them, the external and internal ones membraneous and the middle muscular. The internal coat too, is lined with a sort of a velvety, downy substance, like one side of canton flannel, and is as wrinkled as an old-fashoned washing-board.

It was a long time a matter of doubt and uncertainty how this curiously constructed machine performed the labor it was called upon to do; exhibiting only another instance of how these who have gone before us groped in the dark, and went purely by guess. The Ancient Doctors, led by the Greek EMPEDOCLES, and HIPPOCRATES, those old-fashioned chaps who dissected dogs, monkeys and sheep to find out how man was constructed; these people, thought the stomach was a charnel-house where food was putrified before it was transmitted to other portions of the system, that it rotted as seed does in the ground, before it put on new shape; others contended that the food was ripened by the heat of the stomach in a similar manner as fruit is ripened by the summer sun; still others put these two together, and argued that digestion was only fermentation; while still another class insisted upon it that the stomach was only a mill, where the muscular coatings, like a great mill-stone, ground the food with a pressure of hundreds of thousands of pounds! If this latter theory had been true, it seems to me that digestion would only have been another name for colic!

But this nor any of the other theories were correct.

A man by the name of CHESELDEN guessed at the notion that there was a powerful liquid which did the work of digestion. CHESELDEN was not a Yankee, but his guess, taken hold of and investigated by such men as HALLER, REAUMER, SPALLANZI and other celebrated physiologists, turned out to be a lucky, because the true one.

This liquid is called *gastric juice*, and is one of the most remarkable agents known in nature. It pours itself into the stomach at about the rate of one pound in every twenty-four hours.

With this agent, the stomach closes its doors, calls all the heat from the other portions of the body that can be spared and goes to work bravely on whatever is offered to it.

I shall take occasion, in another paper, to speak of some singular performances of this organ, and its agent the *gastric juice*, and trust to be able to show that the stomach is entitled to more of our respect and consideration than it has heretofore received.

